# A first structural model for covalent dimerization of S100 proteins

**DOI:** 10.1107/S2053230X26002992

**Published:** 2026-04-14

**Authors:** Maria Demou, Laure Yatime

**Affiliations:** a Laboratory of Pathogens and Host Immunity (LPHI) Université de Montpellier, INSERM, CNRS Montpellier France; Bristol-Myers Squibb, USA

**Keywords:** S100 proteins, cysteines, covalent homodimerization, disulfide crosslinking, RAGE receptors

## Abstract

Extracellular S100 proteins act as alarmins and trigger pro-inflammatory signaling cascades by activating cognate cell-surface receptors such as the receptor for advanced glycation end-products (RAGE), thereby contributing to both normal and pathological inflammation depending on the physiological context. These ligand–receptor interactions occur in an oxidative environment that is known to induce post-translational modifications, notably on the cysteine residues present in S100 proteins, giving rise to disulfide-crosslinked S100 species. The fine molecular architecture of these S100 covalent assemblies and their impact on the interaction of S100 with RAGE remains poorly characterized, as most *in vitro* studies employ cysteine variants or reducing conditions. In this study, a thorough analysis of cysteine conservation within the whole S100 family shows an enriched presence of cysteines in the second half of helix H4, with a hotspot for cysteine occupancy at position 84. Following the introduction of a cysteine at this conserved position in S100A6, SDS–PAGE analysis under nonreducing conditions shows a noteworthy amount of covalent S100A6 Y84C dimer in solution, and the structural analysis of the resulting complex with the RAGE ectodomain reveals the formation of a covalent Cys84–Cys84 linkage between the two S100A6 protomers, thus stabilizing the dimeric conformation of RAGE-bound S100A6. Modeling of other S100 proteins that naturally bear a Cys84 in the RAGE-bound conformation suggests that this covalent S100 dimer architecture may be adopted by other members of the family previously reported to form disulfide-crosslinked species. Altogether, these findings provide a first possible model for S100 covalent homodimerization that is fully compatible with RAGE binding.

## Abstract 2

The first molecular description of a disulfide-crosslinked S100 homodimer is revealed by the crystallographic structure of an S100A6 variant bound to the receptor for advanced glycation end-products (RAGE). Sequence-conservation analyses and computational modeling suggest that this covalent architecture may be adopted by other S100 proteins, thus representing a conserved mode of covalent dimerization within the S100 family.

## 1. Introduction

S100 proteins are small calcium-binding proteins from the EF-hand superfamily. They are found almost exclusively in vertebrates, where they exert pleiotropic functions, both intracellularly and extracellularly (Gonzalez *et al.*, 2020 ▸). Inside cells, they regulate calcium-dependent processes linked to calcium homeostasis, metabolism, cell proliferation, motility or cytoskeleton rearrangement (Donato *et al.*, 2013 ▸). Outside cells, several members act as immune-activating alarmins (Bertheloot & Latz, 2017 ▸) by binding to cognate cell-surface receptors such as the receptor for advanced glycation end-products (RAGE; Heizmann *et al.*, 2007 ▸; Leclerc *et al.*, 2009 ▸), Toll-like receptors (Möller *et al.*, 2023 ▸), basigin (Hibino *et al.*, 2013 ▸), neuroplastin (Sakaguchi *et al.*, 2016 ▸), SIRL-1 (Rumpret *et al.*, 2021 ▸), CD33 (Chen *et al.*, 2013 ▸), CD68 (Okada *et al.*, 2016 ▸) or CD69 (Lin *et al.*, 2015 ▸), through which they trigger downstream pro-inflammatory signaling cascades. Several S100 proteins also take part in the antimicrobial response, thanks to their ability to sequester metal ions with high affinity, a property that leads to nutritional immunity (Zackular *et al.*, 2015 ▸). The functional versatility of S100 proteins is thought to arise from their propensity to form distinct assemblies that may exert distinct biological functions (Russo *et al.*, 2022 ▸). Hence, most S100 proteins form dimers as the minimal functional unit; they can also arrange into higher order oligomers, such as tetramers, hexamers or octamers (Heizmann, 2002 ▸; Moroz *et al.*, 2002 ▸; Ostendorp *et al.*, 2007 ▸; Figueira *et al.*, 2022 ▸). Furthermore, they can form heterocomplexes, such as the pro-inflammatory S100A8/A9 heterodimer. Most importantly, their tertiary and quaternary architecture can also be modulated in response to the binding of divalent cations or following post-translational modifications such as phosphorylation, nitrosylation or disulfide-bond formation (Bajor *et al.*, 2016 ▸; Ecsédi *et al.*, 2018 ▸; Leukert *et al.*, 2006 ▸; Ma *et al.*, 2007 ▸).

The impact of redox conditions on the structure–function relationships of S100 proteins is still lacking in-depth investigation, despite the fact that these proteins undergo a drastic environmental change upon their relocalization from the reduced intracellular compartment to the highly oxidative extracellular space, which is even more oxidative in an inflammatory context marked by the abundant production of reactive oxygen species. Interestingly, a few reports have shown that the oxidation of S100 proteins can lead to the formation of disulfide-crosslinked species, both *in vitro* and *in vivo*, and that such a process may serve as a regulatory switch for their alarmin function. For instance, oxidation of S100A8/A9 leads to the formation of a covalent heterodimer through the linkage of Cys3 of S100A9 to Cys42 of S100A8, resulting in a protein with a higher sensitivity to proteolytic degradation (Stephan *et al.*, 2018 ▸), which dampens the high inflammation sustained by the noncovalent assembly (Hoskin *et al.*, 2019 ▸; Lim *et al.*, 2011 ▸), although recent studies have attributed some anti-inflammatory properties to the noncovalent S100A8/A9 tetramer (Lin *et al.*, 2015 ▸). Oxidized S100A4 also assembles into disulfide-crosslinked oligomers that fail to interact with protein phosphatase 5 (PP5) and further prevent PP5 activation by S100A1, thereby modulating apoptosis under oxidative stress conditions (Tsuchiya *et al.*, 2014 ▸). Of importance, the interaction of S100 proteins with RAGE may also be modulated by cysteine-dependent oxidative processes. Hence, the oxidation of S100B generates disulfide-crosslinked octamers that display enhanced binding to RAGE and can induce RAGE-dependent secretion of vascular endothelial growth factor (VEGF), unlike noncovalent S100B dimers (Ma *et al.*, 2007 ▸).

These covalent assemblies most probably represent only a minor pool within the extracellular population of S100 proteins. Current evidence nevertheless suggests they may have some importance for the extracellular functions of S100, including their interaction with effectors such as RAGE, which could offer potential value as therapeutic targets in anti-inflammatory applications. The quaternary architecture of these disulfide-crosslinked species has, however, not yet been characterized at the fine molecular level, which precludes the design of inhibitors specifically targeting these forms. Such a lack is partly inherent to the fact that many *in vitro* studies are performed with recombinant S100 proteins purified under reducing conditions or in which cysteines have been mutated to serine residues (Harman *et al.*, 2022 ▸; Mohan *et al.*, 2013 ▸; Nakashige *et al.*, 2017 ▸; Malashkevich *et al.*, 2008 ▸). As we could not manage to obtain the structure of a full complex between RAGE ectodomain and a S100 protein naturally bearing a cysteine at position 84, we here address this question by examining an engineered variant of S100A6. Following the identification of a conserved cysteine at position 84 that is known to contribute to the covalent homodimerization of other S100 proteins, we engineered an S100A6 Y84C variant and determined the crystallographic structure of its complex with the RAGE ectodomain. This structure reveals that the RAGE-bound S100A6 variant forms a covalent homodimer through Cys–Cys disulfide crosslinking, thus providing a first atomic model to describe the quaternary organization of covalent S100 dimers. Based on computational modeling of other S100 proteins known to bear a cysteine at this position, we discuss the possibility that this architecture may be adopted by other RAGE-binding S100 proteins known to form covalent assemblies.

## 2. Methods

### 2.1. Sequence alignments and conservation analysis

**Figure 1:**
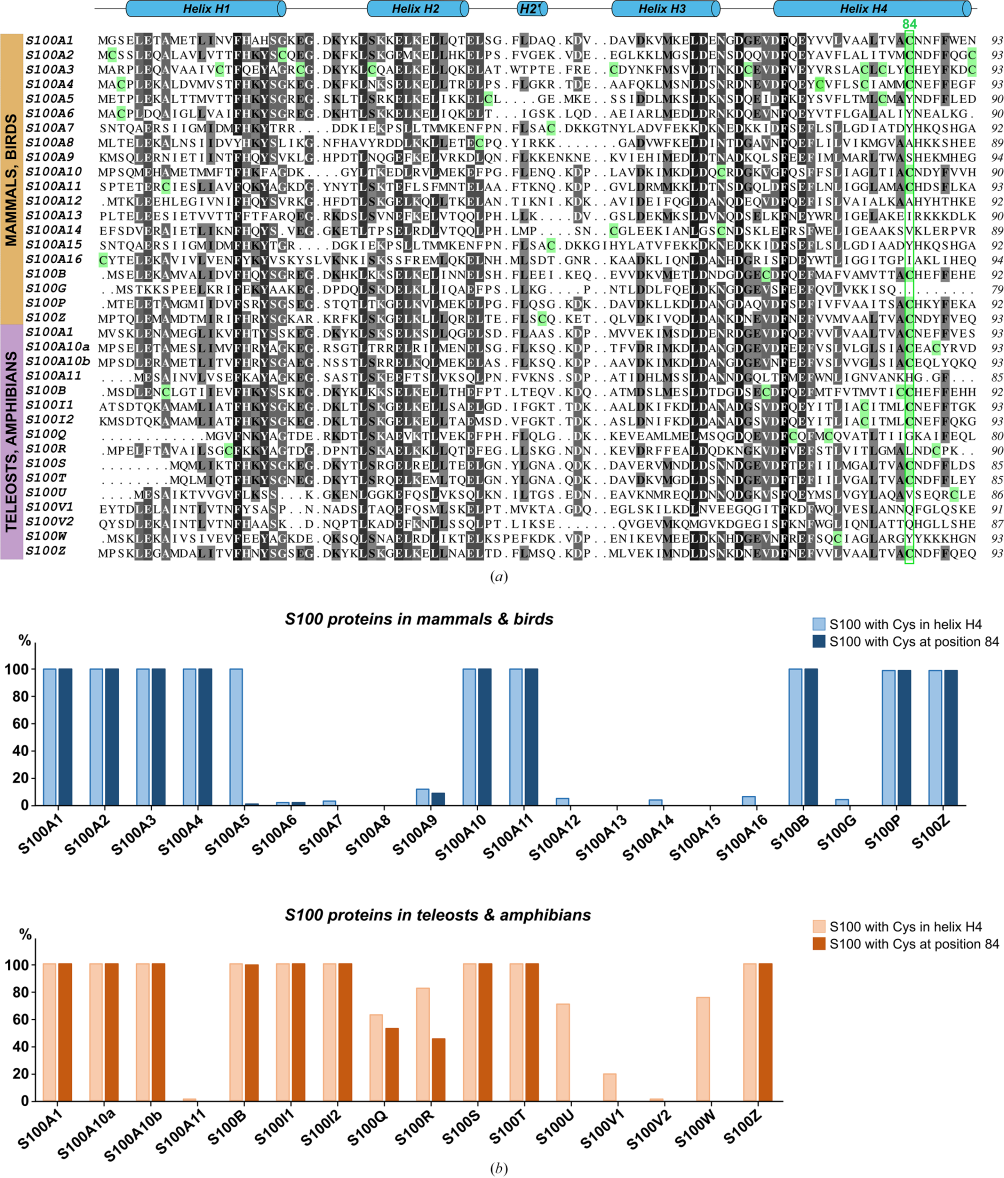
Cysteine conservation within the S100 family. (*a*) Sequence alignment of the 36 distinct representative S100 protein sequences. Human proteins were chosen as representative of mammalian/bird S100 proteins and zebrafish (*Danio rerio*) proteins for teleost/amphibian/reptile S100 proteins, except for S100Q and S100R which are from *Dichotomyctere nigoviridis*. The secondary-structure elements classically found in the S100 fold are shown above the alignment. Cysteine residues are boxed in green and the hotspot position for cysteine conservation (84) is highlighted with a green frame. (*b*) Percentage of sequences retrieved after protein *BLAST* on each unique sequence displayed in (*a*) that contain a cysteine in helix H4 and/or at position 84.

Human proteins were used as representatives of the 20 distinct mammalian/bird S100 proteins and zebrafish (*Danio rerio*) proteins were used as representatives of the 16 distinct teleost/amphibian/reptile S100 proteins, except for S100Q and S100R, which used sequences from green spotted puffer fish (*Dichotomyctere nigroviridis*). All 36 distinct representative sequences were aligned in *Clustal Omega* (Sievers *et al.*, 2011 ▸) and colored according to sequence conservation using *ALINE* (Bond & Schüttelkopf, 2009 ▸; Fig. 1 ▸*a*). Analysis of cysteine conservation for each representative S100 protein sequence was performed by conducting a search against a nonredundant protein database using *BLASTp* from the NCBI server (Altschul *et al.*, 1990 ▸) and manually analyzing the first 100 protein sequences retrieved for the presence of a cysteine residue in the region corresponding to helix H4 and at position equivalent to 84 in human S100A6.

### 2.2. Generation of the S100A6 Y84C variant

The S100A6 Y84C variant was generated by PCR-based site-directed mutagenesis from the pETM11:S100A6 construct previously used to express wild-type (WT) S100A6 (Yatime *et al.*, 2016 ▸), using the following anti-complementary primers bearing the mutation to introduce: forward primer 5′-GGGGCCTTGGCTCTCATATGTAATGAAGCTCTG-3′ and reverse primer 5′-CAGAGCTTCATTACATATGAGAGCCAAGGCCCC-3′. The PCR reaction was performed using Hot Flex Phusion DNA polymerase (New England Biolabs) according to the manufacturer’s instructions.

### 2.3. Expression and purification of S100A6 Y84C variant and RAGE ectodomain

Expression and purification of the recombinant S100A6 Y84C protein was conducted according to previously described procedures for the WT protein (Yatime, 2019 ▸; Yatime *et al.*, 2016 ▸). For the final purification step on size-exclusion chromatography (SEC; 24 ml Superdex 75 Increase column from Cytiva), the protein was eluted in 20 m*M* Tris–HCl pH 7.5, 150 m*M* NaCl, 5 m*M* CaCl_2_ to obtain the Ca^2+^-bound form. Expression and purification of the human RAGE VC1C2 ectodomain (RAGE-VC1C2; residues Ala23–Pro323) was performed as described previously (Yatime & Andersen, 2013 ▸). The protein eluted from the final cation-chromatography step (9 ml Source 15S column from Cytiva) in 40 m*M* Tris–HCl pH 7.5, 450 m*M* NaCl. After SDS–PAGE analysis, the fractions of interest for both proteins were pooled and flash-frozen in liquid nitrogen for storage at −80°C.

### 2.4. Crystallization of the RAGE–S100A6 Y84C complex

Crystallization experiments were carried out using commercial screens from Hampton Research and Molecular Dimensions. Crystals of the RAGE–S100A6 Y84C complex were grown and cryoprotected as described in Table 1 ▸ and were then flash-cooled in liquid nitrogen.

### 2.5. Data collection, structure determination and model refinement

A complete dataset extending to 2.35 Å resolution (Table 2 ▸) was collected at 100 K on the ID30A-1 beamline at ESRF, Grenoble, France. The dataset was processed with *XDS* (Kabsch, 2010 ▸), revealing *I*222 symmetry for the RAGE–S100A6 Y84C crystals, with one molecule of RAGE and one molecule of S100A6 per asymmetric unit, as for the WT complex. The structure was solved by molecular replacement (MR) in *Phaser* (McCoy *et al.*, 2007 ▸), using the structure of the WT complex as a search model (PDB entry 4p2y; Yatime *et al.*, 2016 ▸). Refinement of the model was carried out by alternating cycles of manual rebuilding in *Coot* (Emsley *et al.*, 2010 ▸) and refinement with *phenix.refine* (Adams *et al.*, 2010 ▸) using individual ADP and TLS parameterization. The refinement statistics are reported in Table 3 ▸ and the quality of the model was assessed with *MolProbity* (Davis *et al.*, 2007 ▸). Single omit map generation was performed with *phenix.refine* using simulated annealing after removing residues 83–85 from S100A6 in the input model. All figures were made with the *PyMOL* molecular-graphics system version 0.99rc6 (DeLano Scientific).

### 2.6. Three-dimensional modeling

Three-dimensional models for S100A4 and S100B covalent dimers were generated using *RosettaCM* (Song *et al.*, 2013 ▸), specifying the RAGE-bound S100A6 WT dimer as a custom template (PDB entry 4p2y; Yatime *et al.*, 2016 ▸).

## 3. Results and discussion

### 3.1. The second half of helix H4 in S100 proteins contains conserved Cys residues

Despite the potentially important role of cysteines in modulating the biological function of S100 proteins, no in-depth analysis of their conservation on the scale of the whole S100 family has yet been reported. Mammalian and bird S100 proteins encompass up to 20 distinct members, as represented by the sequences of human S100 proteins in Fig. 1 ▸(*a*). More recent data on teleost fish, amphibians and reptiles revealed the presence of a distinct set of S100 proteins in these vertebrates (Kraemer *et al.*, 2008 ▸), showing a rather distant phylogenetic lineage compared with mammalian orthologs, and counting up to 16 members, as illustrated by the sequences of the proteins from zebrafish (*Danio rerio*) and green spotted puffer fish (*Dichotomyctere nigroviridis*). Alignment of these 36 distinct S100 sequences (Fig. 1 ▸*a*) already highlights an enriched presence of cysteines in the second half of helix H4, with the strongest cysteine occupancy found at position 84 (human S100A6 numbering).

For a more robust analysis of cysteine conservation, protein *BLAST* was performed against nonredundant protein sequences for each unique sequence listed in Fig. 1 ▸(*a*), the first hundred most relevant homologs were retrieved and the number of sequences encompassing a cysteine in helix H4 and/or at position 84 was counted manually. As shown in Fig. 1 ▸(*b*), half of the mammalian/bird S100 proteins always contain a cysteine in H4 and almost 75% of teleost/amphibian S100 sequences similarly do. Importantly, more than half of all the sequences analyzed bear a cysteine at position 84, and when such a residue is present in the representative sequence displayed in Fig. 1 ▸(*a*), it is present in all homologous sequences. This suggests that position 84 is a hotspot for cysteine conservation, hinting at a potential role of this residue in the function of S100 proteins.

### 3.2. Introduction of a cysteine at the conserved position 84 stabilizes RAGE-bound S100A6 through covalent homodimerization

**Figure 2:**
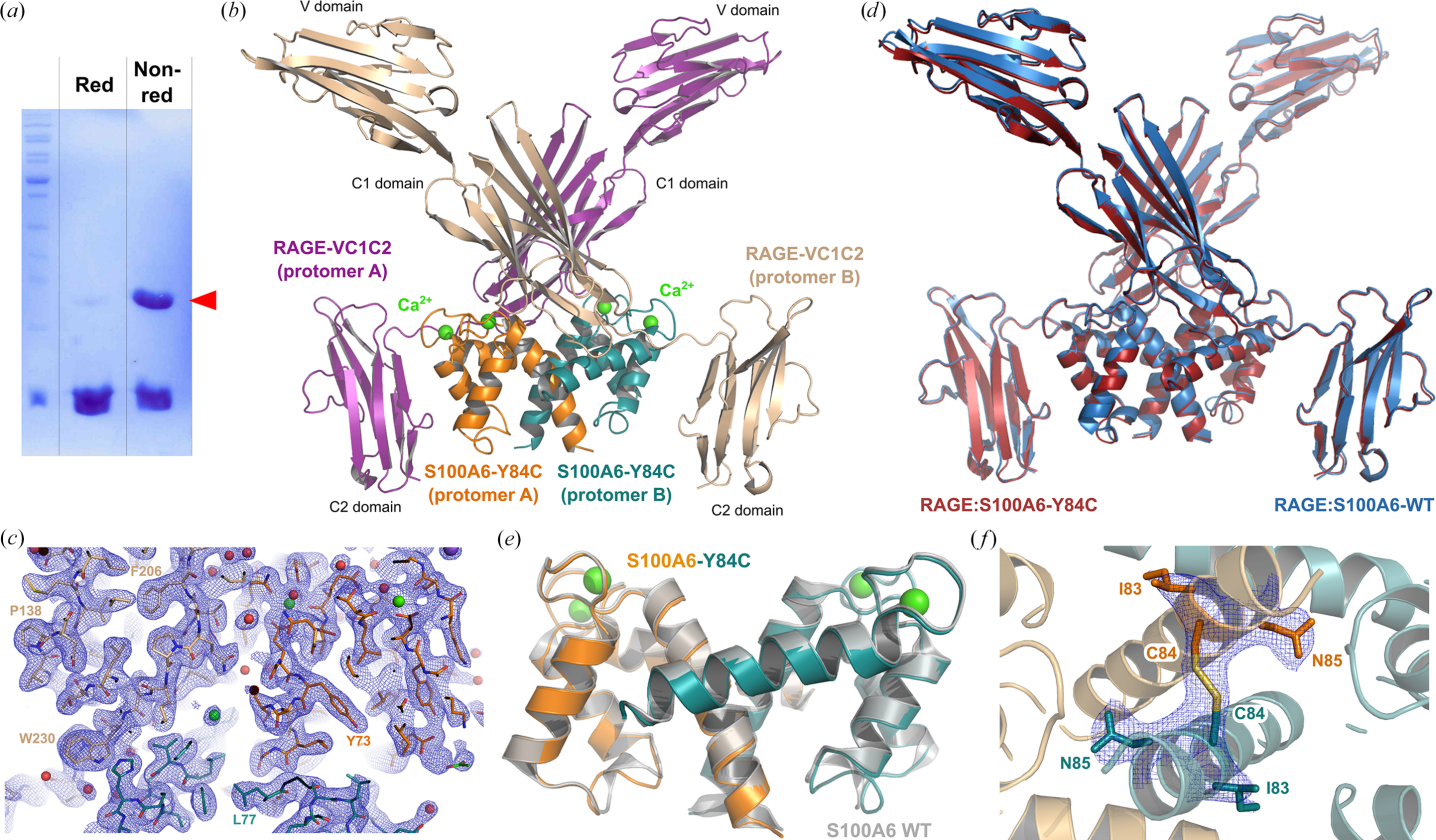
Introduction of Cys84 yields a disulfide-crosslinked S100A6 dimer. (*a*) SDS–PAGE analysis of the S100A6 Y84C variant under reducing (Red) and nonreducing (Non-red) conditions. A band at double the size of that of the S100A6 monomer is visible under nonreducing conditions, indicating that the protein readily forms disulfide-crosslinked dimers in solution. (*b*) Crystallographic structure of the RAGE–S100A6 Y84C complex at 2.35 Å resolution. The biological assembly encompasses two molecules of RAGE bound to one S100A6 homodimer (2:2 complex). (*c*) Overlay of the refined atomic model with the final electron-density map (2*F*_o_ − *F*_c_ map, blue mesh, contoured at 1 r.m.s.d). (*d*) The RAGE–S100A6 Y84C complex (red) superimposes extremely well with the RAGE–S100A6 WT complex (blue). (*e*) Superimposition of the RAGE-bound S100A6 Y84C dimer (orange and teal) with the RAGE-bound S100A6 WT dimer (gray). Both adopt the same dimeric conformation. (*f*) Zoom on the C-terminal helices (H4) of both S100 protomers in the RAGE-bound S100A6 Y84C homodimer. The two protomers are covalently linked via an intermolecular disulfide bridge (Cys84–Cys84). The single simulated-annealing omit map calculated in *phenix.refine* after removing S100A6 residues Ile83–Asn85 in the model is displayed as blue mesh around this region (contoured at 1 r.m.s.d), allowing visualization of the clearly defined density for the S—S bond.

Interestingly, several of the human S100 proteins bearing a cysteine at position 84 are known to bind to the RAGE receptor, including S100A1, S100A2, S100A4, S100B and S100P. The formation of disulfide-crosslinked species has even been reported to enhance RAGE binding for S100B (Ma *et al.*, 2007 ▸). However, the molecular rationale for such an effect is still lacking due to the absence of a structural description of these covalent species. To date, only one S100 protein has been crystallized in a full complex with the RAGE entire ecto­domain: S100A6 (Yatime *et al.*, 2016 ▸). Despite extensive efforts, we did not manage to obtain the structure of a full-length complex with the RAGE ectodomain for other S100 proteins, including those naturally carrying a cysteine at position 84, such as S100B or S100A4. To gain insights into how the presence of a cysteine at this position may affect the architecture of S100s and potentially modulate their inter­action with RAGE, we therefore generated the Y84C variant in S100A6 (S100A6 Y84C). As shown by SDS–PAGE analysis under nonreducing conditions, a noteworthy fraction of the protein readily forms disulfide-crosslinked dimers in solution (Fig. 2 ▸*a*). We next co-crystallized it with the RAGE full-length ectodomain (fragment VC1C2). Crystals of the complex that have symmetry consistent with space group *I*222 were obtained and diffracted to 2.35 Å resolution. Phasing was achieved using molecular replacement with the structure of the WT complex as search template (PDB entry 4p2y; Yatime *et al.*, 2016 ▸). The refined atomic model and final electron-density map are displayed in Figs. 2 ▸(*b*) and 2 ▸(*c*). The variant complex adopts a 2:2 stoichiometry and is highly similar to the WT complex, with an overall root-mean-square deviation (r.m.s.d.) on C^α^ atoms of 0.4 Å between the two structures (Fig. 2 ▸*d*). The highest r.m.s.d. values are observed in a few discrete loop regions connecting β-strands in immunoglobulin domains V and C1 in the RAGE molecule, and in the hinge region connecting helices H2 and H3 in S100A6 (Supplementary Fig. S1), all of these being intrinsically quite flexible.

In the variant complex, S100A6 Y84C adopts the same homodimeric conformation as that of RAGE-bound WT S100A6 (Fig. 2 ▸*e*), *i.e.* helices H1 and H4 from the first S100A6 protomer are arranged in an antiparallel manner with the same helices from the second S100A6 protomer. Most interestingly, as highlighted by a closer view of the region of the atomic model near position 84 within S100A6, the two mutated Cys84 residues from each protomer are in such close proximity that a disulfide bridge is formed between them, thus giving rise to a disulfide-crosslinked S100A6 Y84C dimer (Fig. 2 ▸*f*). The presence of this S–S linkage is not due to a modeling bias since it is visible in the single simulated-annealing omit map calculated from a model in which residues 83–85 of S100A6 have been omitted (Fig. 2 ▸*f*, blue mesh). Thus, at least for S100A6, the presence of a cysteine at the conserved position 84 is not only compatible with RAGE binding but also further stabilizes the RAGE-bound S100 dimer through covalent linkage of the two S100 protomers.

### 3.3. Other S100 proteins may form covalent dimers through Cys84–Cys84 bridging

**Figure 3:**
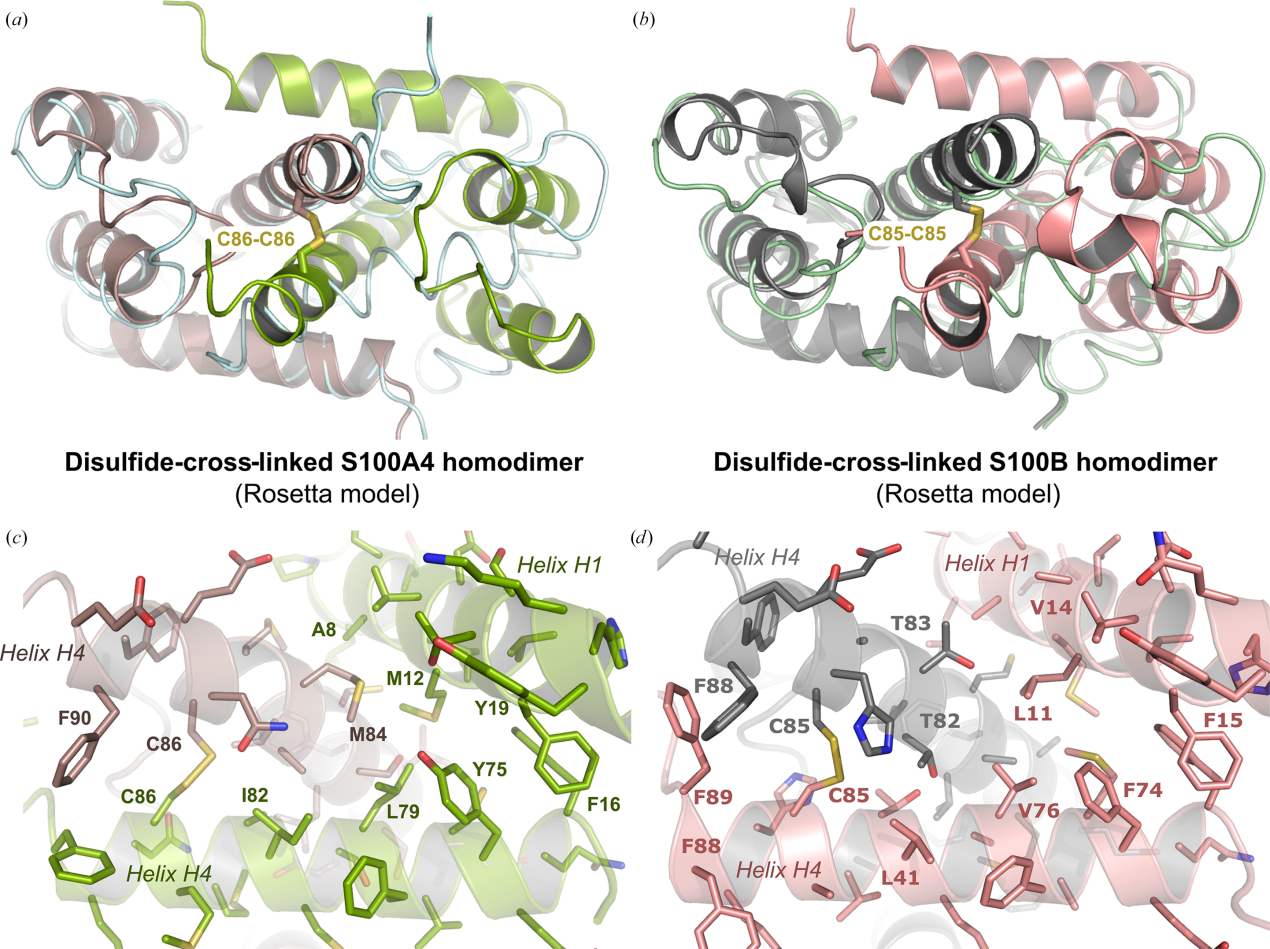
A first possible model for S100 disulfide-crosslinked homodimers. (*a*) *Rosetta*-based modeling of the S100A4 homodimer (PDB entry 2q91; Malashkevich *et al.*, 2008 ▸) in the RAGE-bound S100A6 conformation yields a disulfide-crosslinked dimer through a Cys86–Cys86 linkage. The structure of the noncovalent S100A4 homodimer (PDB entry 2q91) is shown as light blue cartoon for comparison. (*b*) *Rosetta*-based modeling of an S100B homodimer (PDB entry 3d0y; Ostendorp *et al.*, 2011 ▸) in the RAGE-bound S100A6 conformation yields a disulfide-crosslinked dimer through Cys85–Cys85 linkage. The structure of the non­covalent S100B homodimer (PDB entry 3d0y) is shown as a light green cartoon for comparison. (*c*, *d*) Zoom on the interface within the covalent S100A4 and S100B homodimers. In both cases, a network of aromatic and aliphatic residues on helices H1 and H4 maintains the interface through hydrophobic interactions.

While Cys84 is not naturally present in S100A6, a cysteine in the second half of helix H4 is found in at least ten distinct S100 proteins known to bind to RAGE, including S100B, S100A2, S100A4 and murine S100A9, that were all reported to form disulfide-crosslinked dimers (Deshpande *et al.*, 2000 ▸; Ma *et al.*, 2007 ▸; Signor *et al.*, 2021 ▸; Tsuchiya *et al.*, 2014 ▸). Thus, under oxidative conditions, helix H4 cysteines might promote the formation of covalent S100 dimers. As a first step towards the corroboration of this hypothesis, we used the *Robetta* server (Song *et al.*, 2013 ▸) to model S100A4 and S100B in the RAGE-bound S100A6 dimeric conformation. In both cases this yielded a disulfide-crosslinked dimer, with an S–S bridge formed between the two conserved cysteines at position 84 (85 in S100B and 86 in S100A4) from each S100 protomer (Figs. 3 ▸*a* and 3 ▸*b*). The resulting dimeric interfaces cover large surfaces, with total buried areas of 1440 and 1495 Å^2^ for S100A4 and S100B, respectively. Furthermore, they are held in place by an extended network of hydrophobic interactions (Figs. 3 ▸*c* and 3 ▸*d*), as in the noncovalent S100 dimers (Malashkevich *et al.*, 2008 ▸; Ostendorp *et al.*, 2011 ▸), and several hydrogen bonds may further strengthen the interfaces, providing the presence of a complete hydration shell that is not represented in the current models. Thus, these covalent assemblies are physically plausible and appear to be as stable as the noncovalent assemblies.

As an argument in favor of this model, Cys85 was shown to be important for the formation of S100B covalent species (Scotto *et al.*, 1998 ▸). However, this residue is deeply buried within the S100B protomer (PDB entry 3d0y; Ostendorp *et al.*, 2011 ▸) in the noncovalent assembly and cannot engage in interprotomer Cys–Cys interactions. The formation of covalent dimers through disulfide crosslinking of this cysteine would therefore require an important rearrangement of the S100B quaternary architecture, while preserving its tertiary fold to maintain biological interactions. The structure of the RAGE-bound S100A6 Y84C dimer may thus represent one possible quaternary organization for S100 disulfide-crosslinked dimers that offers the advantage of retaining the same S100 surfaces exposed to the solvent as in the noncovalent dimers, thereby still enabling interaction with binding partners such as RAGE. Obviously, other types of covalent assemblies may exist as other cysteine residues at distinct positions have been implicated in disulfide crosslinking of S100 proteins.

## 4. Conclusion

In conclusion, this study provides a structural description of a first possible model for S100 covalent dimerization and identifies a conserved cysteine in the second half of helix H4 as a potential switch to drive the covalent linkage of various S100 proteins known to bind RAGE. The demonstration that such covalent S100 dimers exist *in vivo* and the characterization of their precise architecture will be the object of further investigation, in order to provide useful insights on the design of more selective S100 inhibitors for anti-inflammatory applications.

## Supplementary Material

PDB reference: complex between human RAGE ectodomain and S100A6 variant Y84C, 9s2x

Supplementary Figure S1. DOI: 10.1107/S2053230X26002992/rf5050sup1.pdf

## Figures and Tables

**Table 1:** Crystallization of the RAGE–S100A6 Y84C variant complex

Method	Vapor diffusion in sitting drops
Plate type	SWISSCI MRC-2
Temperature (°C)	4
Protein concentration	6 mg ml^−1^ (for RAGE-VC1C2), twofold molar excess of S100A6 Y84C
Buffer composition of protein solution	20 m*M* Tris–HCl pH 7.5, 200 m*M* NaCl, 5 m*M* CaCl_2_
Composition of reservoir solution	0.2 *M* zinc acetate, 0.1 *M* sodium cacodylate pH 6.5, 9% 2-propanol + 2 m*M* NDSB-221 as additive
Volume and ratio of drop	2 µl (0.9 µl protein + 0.9 µl reservoir + 0.2 µl additive)
Volume of reservoir (µl)	50
Composition of the cryoprotectant	0.2 *M* zinc acetate, 0.1 *M* sodium cacodylate pH 6.5, 40% 2-propanol
Drop setting	Manually set up
Seeding	No

**Table 2:** Data collection and processing for the RAGE–S100 Y84C variant complex Values in parentheses are for the highest resolution shell.

Diffraction source	ID30A-1, ESRF
Wavelength (Å)	0.9654
Temperature (K)	100
Detector	PILATUS3 6M
Crystal-to-detector distance (mm)	288
Total rotation range (°)	180
Rotation per image (°)	0.2
Space group	*I*222
*a*, *b*, *c* (Å)	75.65, 113.01, 139.51
α, β, γ (°)	90, 90, 90
Mosaicity (°)	0.229
Resolution range (Å)	50–2.35 (2.42–2.35)
Total No. of reflections	160492 (13848)
No. of unique reflections	25103 (2107)
Completeness (%)	99.1 (100)
Multiplicity	6.4 (6.6)
〈*I*/σ(*I*)〉 from merged data	15.37 (2.10)
CC_1/2_	99.9 (79.3)
*R*_meas_ (%)	8.1 (88.4)
Overall *B* factor from Wilson plot (Å^2^)	48

**Table 3:** Structure refinement for the RAGE–S100 Y84C variant complex Values in parentheses are for the highest resolution shell.

Resolution range (Å)	43.9–2.35
Completeness (%)	99.1
No. of unique reflections	25097
Final *R*_work_	0.2043
Final *R*_free_	0.2352
Estimated coordinate error (Å)	0.33
No. of non-H atoms	
Protein	2988
Ions	29
Waters	205
Total	3222
R.m.s. deviations from ideality	
Bond lengths (Å)	0.003
Angles (°)	0.6
Average *B* factors (Å^2^)	
Protein	60
Ions	52
Waters	55
Ramachandran plot	
Favored regions (%)	98.4
Outliers (%)	0
Unmodeled/incomplete residues (%)	0
PDB code	9s2x

## Data Availability

Atomic coordinates and structure factors for the structure of the RAGE–S100A6 Y84C complex have been deposited in the Protein Data Bank with accession code 9s2x (https://doi.org/10.2210/pdb9S2X/pdb).
