# Supplementary material for: A first structural model for covalent dimerization of S100 proteins
Source: Acta Crystallogr F Struct Biol Commun. 2026 Apr 14;82(Pt 5):176–83. doi: 10.1107/S2053230X26002992 (PMC13133999; doi:10.1107/S2053230X26002992)
Supplement: Supplementary file 1 [file f-82-00176-sup1.pdf]

Acta Cryst F

STRUCTURAL BIOLOGY COMMUNICATIONS

Volume 82 (2026)

Supporting information for article:

A first structural model for covalent dimerization of S100 proteins

Maria Demou and Laure Yatime

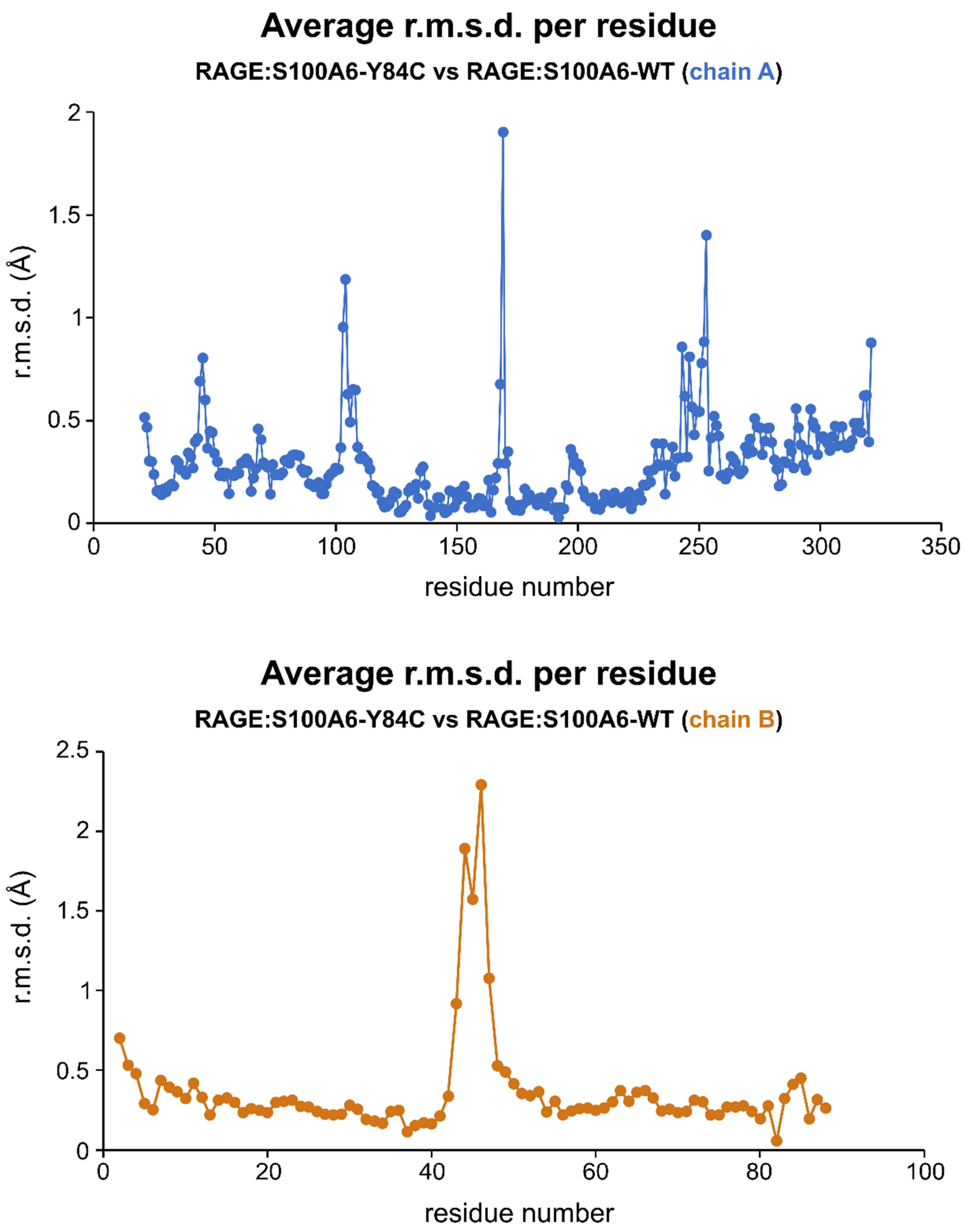

Figure S1 Average root-mean-square deviation (r.m.s.d.) per residue in Å between the structure of the RAGE:S100A6-Y84C variant complex (*this study*) and the RAGE:S100A6-WT complex (PDB_ID 4YBH, (Yatime *et al.*, 2016)). R.m.s.d. values were calculated with COOT (Emsley *et al.*, 2010) following superimposition of the variant complex structure on top of the WT complex structure. The upper and lower panels show the values obtained for chain A (RAGE moiety) and chain B (S100A6 moiety), respectively.
